# Low-dose topiramate and hydrochlorothiazide-associated early acute myopia and angle narrowing: A case report

**DOI:** 10.3389/fmed.2023.1062160

**Published:** 2023-02-08

**Authors:** Chao Wu, Hong Pan, Shijun Feng, Xiaokun Wang, Zhaoqiang Liu, Bojun Zhao

**Affiliations:** ^1^Department of Ophthalmology, Shandong Provincial Hospital, Shandong University, Jinan, China; ^2^Department of Ophthalmology, Shandong Provincial Hospital Affiliated to Shandong First Medical University, Jinan, China

**Keywords:** topiramate, hydrochlorothiazide, angle narrowing, drug–drug interaction, short time

## Abstract

**Purpose:**

To report a unique case of topiramate and hydrochlorothiazide associated with acute myopia and angle narrowing.

**Patients and methods:**

A 34-year-old Asian woman presented with prominent binocular visual acuity decrease 6 h after taking only one dose of 25 mg topiramate, 25 mg hydrochlorothiazide, and 22.4 mg fluoxetine to lose weight. She was subsequently diagnosed with acute bilateral myopia and angle narrowing and was started on topical therapy.

**Results:**

Initial examination revealed a decreased visual acuity of 20/100 bilaterally, an elevated intraocular pressure of 23 mmHg in the right eye and 24 mmHg in the left eye, suprachoroidal effusions, and angle narrowing. After the discontinuation of these drugs and the use of IOP-lowering medication, the patient made full recovery.

**Conclusion:**

We speculate that there is a drug–drug interaction between topiramate and hydrochlorothiazide that may lead to the angle narrowing in a short time and at a low dose. Timely discontinuation of the drug usually leads to complete recovery within days to weeks.

## 1. Introduction

Topiramate was initially used as an antiepileptic but has now become more popular for the prophylaxis of migraine and trigeminal neuralgia. Hydrochlorothiazide is a widely used diuretic. Off-label, both of them have been used for weight loss. Both of them are sulfonamide and its derivatives, which have been reported as causing transient myopia, ciliary body edema, uveal effusions, and secondary acute angle closure glaucoma (aACG) (1–4). However, there is limited literature on the combined use of both drugs that causes adverse effects. Here, we reported a case of a young female patient presenting with bilaterally acute myopia and angle narrowing 6 h after taking only one dose of 25 mg topiramate and 25 mg hydrochlorothiazide to lose weight.

## 2. Case report

**Figure 1:**
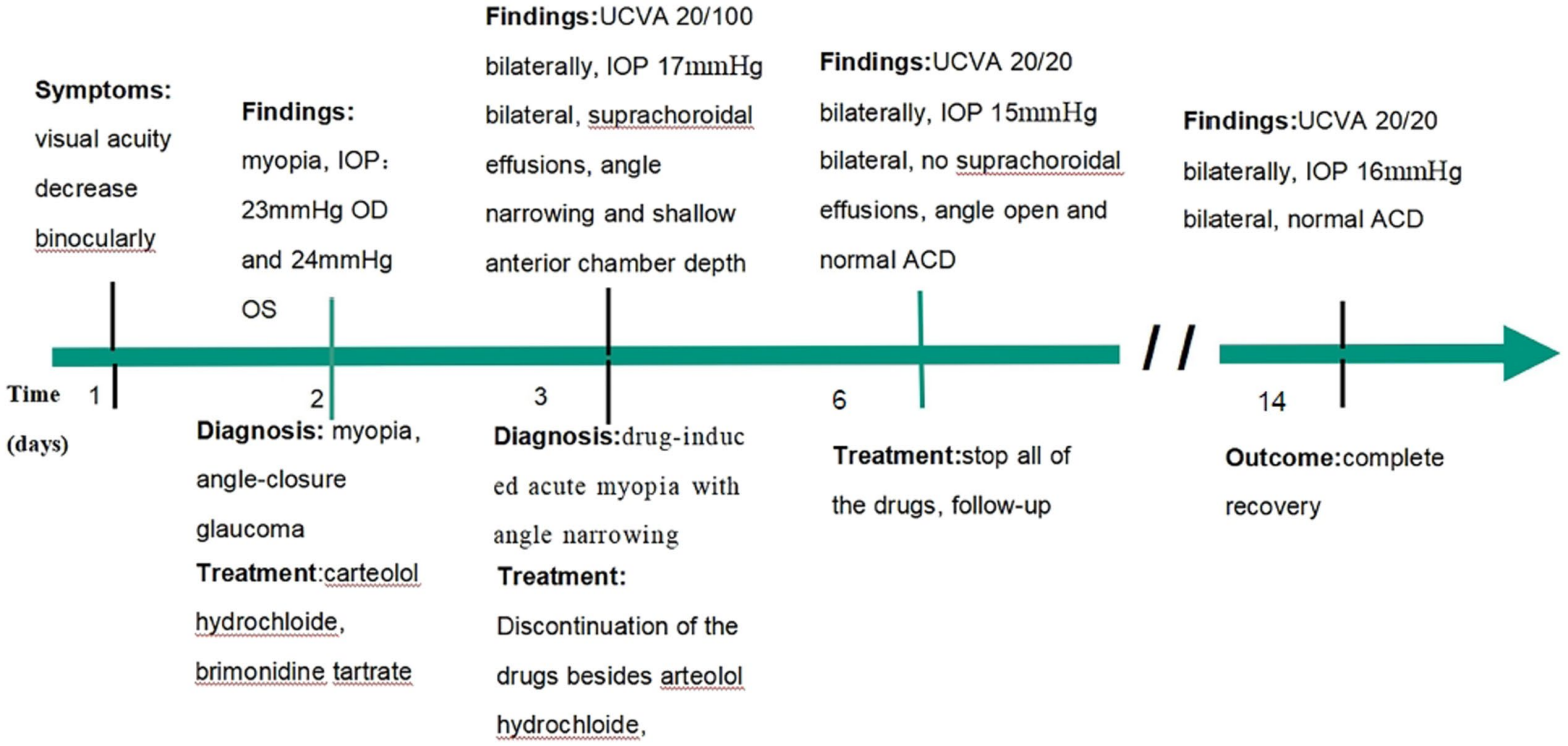
Timeline from start to resolution of acute myopic and angle narrowing induced by topiramate and hydrochlorothiazide.

**Figure 2:**
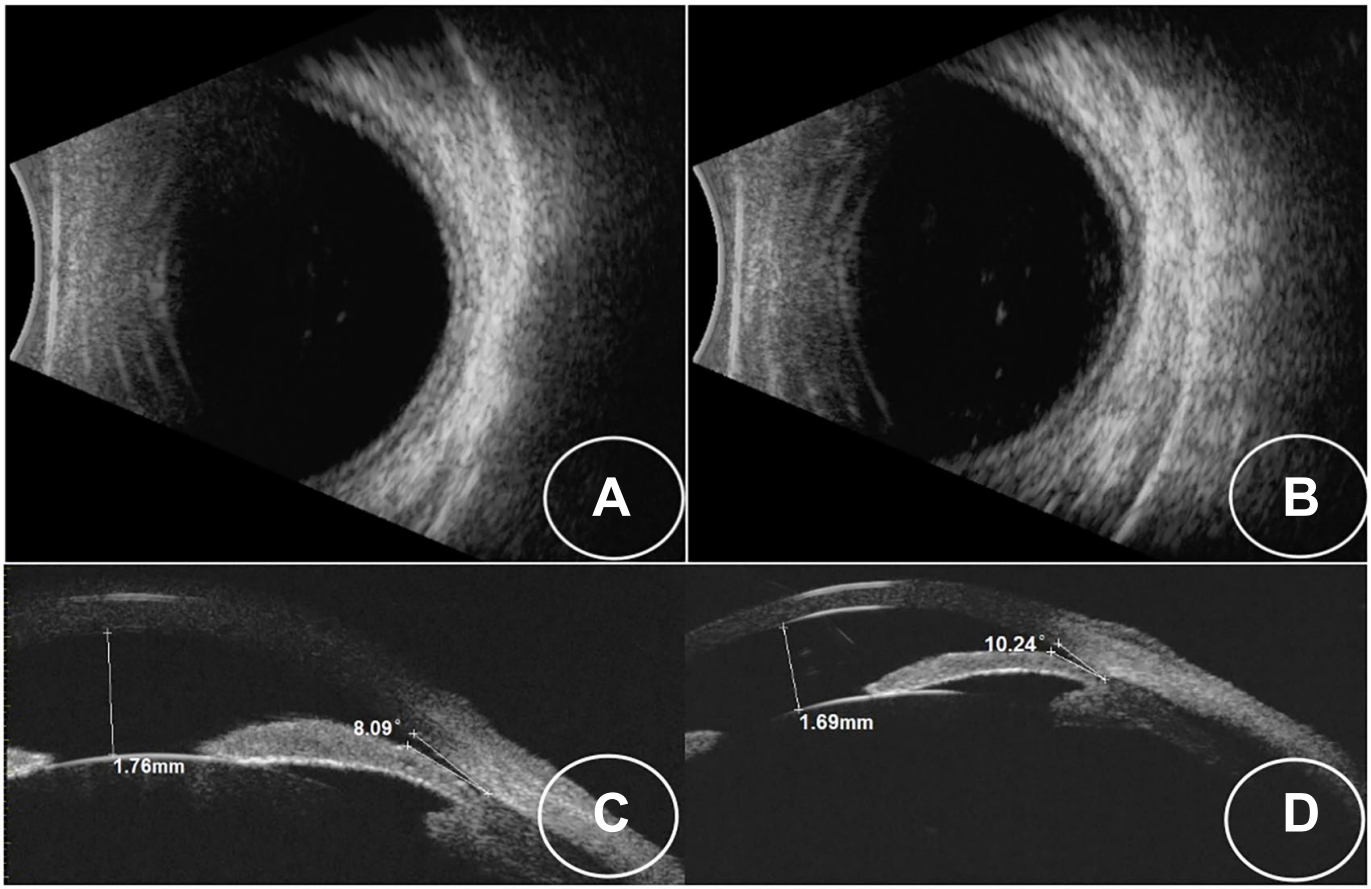
**(A,B)** B-scan images showing choroidal effusion in both eyes. **(C,D)** UBM images showing that the iris-lens diaphragm is pressed against the anterior chamber angle in both eyes.

**Figure 3:**
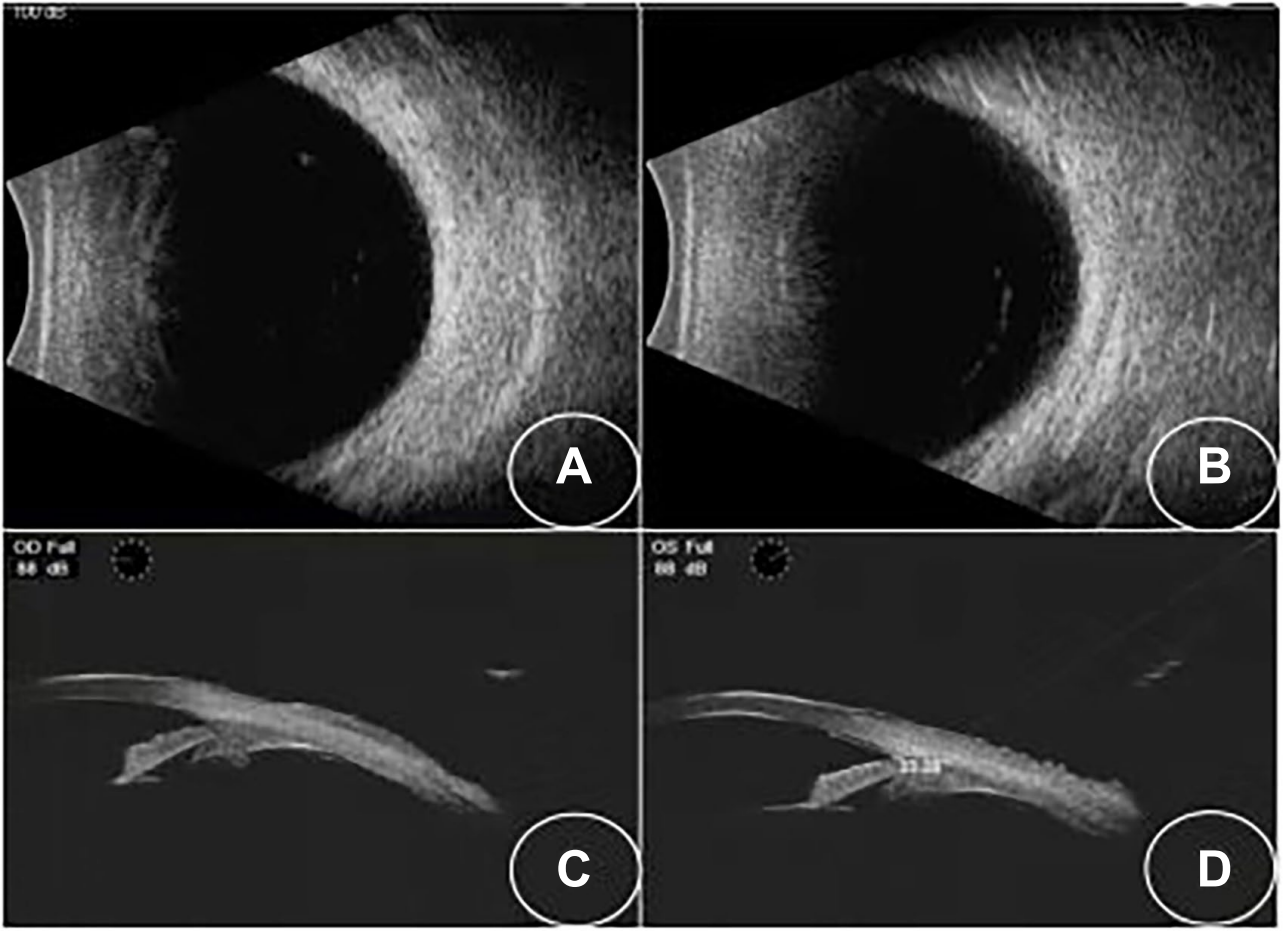
**(A,B)** B-scan showing that the effusions have resolved. **(C,D)** UBM images showing that the angle opened in both eyes.

A 34-year-old Asian woman presented with prominent binocular visual acuity decrease 6 h after taking only one dose of 25 mg topiramate, 25 mg hydrochlorothiazide, and 22.4 mg fluoxetine, and it was the first time for her to take these three drugs. The next day, she saw a local clinician and was told to have acute myopia and high intraocular pressure (IOP; 23 mmHg of the right eye and 24 mmHg of the left eye; Figure 1). She was given carteolol hydrochloride and brimonidine tartrate eye drops to control the elevated IOP. Forty-eight hours after onset, the patient came to our hospital. On examination, her uncorrected visual acuity (UCVA) was 20/100 in both eyes and her IOP was 17 mmHg bilaterally. In addition, her best corrected visual acuity (BCVA) was 20/20 bilaterally with a refractive correction of −3.75 diopters of the right eye and a refractive correction of −4.00 diopters of the left eye. Slit-lamp examination showed bilateral clear cornea, shallow anterior chambers, and equally reactive pupils with a diameter of about 3 mm. There were no signs of inflammation in both eyes. Undilated funduscopic examination findings were normal with a cup-to-disk ratio of 0.3 and a healthy neuroretinal rim in both eyes. A-scan ultrasonography revealed an axial length of 23.7 mm OD and 23.8 mm OS. B-scan ultrasonography revealed suprachoroidal effusion in both eyes (Figure 2). Ultra-biomicroscopy (UBM) showed the narrow angles and revealed that the depth of the central anterior chambers was shallow with 1.71 mm of her right eye and 1.73 mm of her left eye (Figure 2). This young patient did not have any ocular or systemic diseases. Pregnancy was also ruled out. Being a woman of a young age and based on her myopic status, bilateral attacks, equally reactive pupils with a diameter of about 3 mm, and normal fundus appearance, acute primary angle closure glaucoma as well as idiopathic uveal effusion syndrome were less likely, and the diagnosis of bilaterally drug-induced acute myopia with angle narrowing was made. Topiramate, hydrochlorothiazide, fluoxetine, and brimonidine tartrate eye drops were immediately discontinued. Two days later, her UCVA improved to 20/20 in both eyes [autorefractometry sph +0.25 diopter (D) OD, sph +0.25 D + cyl +0.25D * 7° OS]. Slit-lamp examination showed no abnormalities. A-scan ultrasonography revealed an axial length of 23.82 mm OD and 23.91 mm OS. UBM and B scan ultrasound showed no abnormalities in both eyes (Figure 3). Her bilateral IOP was 15 mmHg. She was advised to stop the carteolol hydrochloride eye drops. She took a follow-up after the first application. At the last follow-up, the UCVA of the patient was 20/20 in both eyes and the IOP was 16 mmHg. No abnormalities were shown by the slit-lamp examination.

## 3. Discussion

In our case, the patient took three drugs, but we believed that topiramate and hydrochlorothiazide played the major role. Fluoxetine, one of the selective serotonin reuptake inhibitors (SSRIs), has been reported to cause glaucoma (5). But it is generally believed that SSRIs increase postsynaptic serotonin *via* the desensitization of a rate-limiting enzyme in serotonin generation. Stimulation of the serotonin 5-HT7 receptor leads to iris sphincter relaxation, causing passive mydriasis, as well as increased aqueous humor production, which eventually contributes to aACG (6). However, this was not consistent with our patient because there were no pupillary block matters in our case.

Although the exact mechanism of the adverse effect caused by sulfonamide and its derivatives remains unclear, there are some views. Rhee et al. consider that aACG is attributed to ciliochoroidal effusion and swelling of the ciliary body, causing anterior rotation of the ciliary processes, the narrowness of the ciliary sulcus, and forward displacement of the lens–iris diaphragm (7). Ciliary body effusion also decreases the tension in lens zonules, leading to lens thickening and a myopic shift, manifesting as blurred vision (7). It has been theorized that topiramate or other sulfa medications can induce choroidal expansion by disrupting the blood–ocular barrier through an inflammatory mechanism (8).

Both drugs can cause the adverse effect alone, of which topiramate is most likely because it is the most reported one between the two drugs and the signs of our patients are in accord with the mechanisms of topiramate-induced adverse effects. AACG is particularly common in young adults who are first treated with topiramate (9). However, topiramate was also less likely to cause adverse effects alone. The onset induced by topiramate usually ranges from 1 to 49 days, with a mean of 7 days from the beginning of therapy. When the dose of topiramate is doubled, symptoms happen within hours (10). In contrast, our case was of a low dose and in a short time. Besides, plasma topiramate concentrations can be increased by coadministration of hydrochlorothiazide (11). In addition, thiazide diuretics are one of the possible risk factors for aACG when they are in combination with topiramate (9). Thus, we had reason to believe that there was a drug–drug interaction between the two drugs that may cause the adverse event and shortened the time of the onset.

The management of sulfonamide and its derivatives related to acute myopia and non-pupillary-block angle closure requires consultation with the prescribing physician and cessation of the precipitating medication timely. IOP should be lowered medically, such as with topical β-blockers and prostaglandin analogs. While oral acetazolamideis effective in some cases, some authors argue against its use given that it is a sulfa drug. Cycloplegic agents also help to relax the ciliary muscles, allowing the lens–iris diaphragm to move posteriorly, deepening the anterior chamber and re-opening the angle (10). Topical and/or systemic corticosteroids are often used in cases because uveal effusions are considered to have an inflammatory component (5). Miotics should be avoided as they may worsen angle closure (6). Though peripheral laser iridotomy is not effective in non-pupillary block angle closure, some patients with topiramate-induced AACG who were treated by laser peripheral iridoplasty recovered who had been refractory to treatment with ocular antihypertensives (12). In a word, physicians should be aware of the adverse effect and inform patients of potential risks.

## Data availability statement

The original contributions presented in the study are included in the article/Supplementary material, further inquiries can be directed to the corresponding authors.

## Ethics statement

All procedures performed in studies involving human participants were following the ethical standards of the institutional and/or national research committee and with the ethical standards of the Declaration of Helsinki. Written informed consent was obtained from the individual for the publication of any potentially identifiable images or data included in this article.

## Author contributions

BZ, ZL, and CW: conceptualization, supervision, and writing—review and editing. CW and HP: data curation. CW, HP, SF, and XW: writing—original draft. All authors contributed to the article and approved the submitted version.

## Conflict of interest

The authors declare that the research was conducted in the absence of any commercial or financial relationships that could be construed as a potential conflict of interest.

## Publisher’s note

All claims expressed in this article are solely those of the authors and do not necessarily represent those of their affiliated organizations, or those of the publisher, the editors and the reviewers. Any product that may be evaluated in this article, or claim that may be made by its manufacturer, is not guaranteed or endorsed by the publisher.
